# Turn-taking in cooperative offspring care: by-product of individual provisioning behavior or active response rule?

**DOI:** 10.1007/s00265-017-2391-4

**Published:** 2017-10-17

**Authors:** James L. Savage, Lucy E. Browning, Andrea Manica, Andrew F. Russell, Rufus A. Johnstone

**Affiliations:** 1 Department of Zoology, University of Cambridge, Downing Street, Cambridge, CB2 3EJ UK; 2 Behavioural Ecology Group, Department of Animal Sciences, Wageningen University, De Elst 1, 6708 WD Wageningen, The Netherlands; 3 School of Biological, Earth, and Environmental Sciences, University College Cork, Distillery Fields, North Mall, Cork, T23 TK30 Ireland; 4 Fowlers Gap Arid Zone Research Station, School of Biological, Earth and Environmental Sciences, University of New South Wales, Sydney, 2052 NSW Australia; 5 Centre for Ecology and Conservation, College of Life and Environmental Sciences, University of Exeter, Tremough Campus, Penryn, TR10 9FE UK

**Keywords:** Cooperative breeding, Parental care, Provisioning rules, Reciprocity

## Abstract

**Abstract:**

For individuals collaborating to rear offspring, effective organization of resource delivery is difficult because each carer benefits when the others provide a greater share of the total investment required. When investment is provided in discrete events, one possible solution is to adopt a turn-taking strategy whereby each individual reduces its contribution rate after investing, only increasing its rate again once another carer contributes. To test whether turn-taking occurs in a natural cooperative care system, here we use a continuous time Markov model to deduce the provisioning behavior of the chestnut-crowned babbler (*Pomatostomus ruficeps*), a cooperatively breeding Australian bird with variable number of carers. Our analysis suggests that turn-taking occurs across a range of group sizes (2–6), with individual birds being more likely to visit following other individuals than to make repeat visits. We show using a randomization test that some of this apparent turn-taking arises as a by-product of the distribution of individual inter-visit intervals (“passive” turn-taking) but that individuals also respond actively to the investment of others over and above this effect (“active” turn-taking). We conclude that turn-taking in babblers is a consequence of both their individual provisioning behavior and deliberate response rules, with the former effect arising through a minimum interval required to forage and travel to and from the nest. Our results reinforce the importance of considering fine-scale investment dynamics when studying parental care and suggest that behavioral rules such as turn-taking may be more common than previously thought.

**Significance statement:**

Caring for offspring is a crucial stage in the life histories of many animals and often involves conflict as each carer typically benefits when others contribute a greater share of the work required. One way to resolve this conflict is to monitor when other carers contribute and adopt a simple “turn-taking” rule to ensure fairness, but natural parental care has rarely been studied in sufficient detail to identify such rules. Our study investigates whether cooperatively breeding chestnut-crowned babblers “take turns” delivering food to offspring, and (if so) whether this a deliberate strategy or simply a by-product of independent care behavior. We find that babblers indeed take turns and conclude that part of the observed turn-taking is due to deliberate responsiveness, with the rest arising from the species’ breeding ecology.

**Electronic supplementary material:**

The online version of this article (10.1007/s00265-017-2391-4) contains supplementary material, which is available to authorized users.

## Introduction

Individuals cooperating to rear offspring face several problems when attempting to share provisioning effort efficiently. Firstly, care is costly (Williams 1966), so each individual stands to gain if other carers contribute a greater share of the required provisioning. Secondly, each individual has only partial knowledge of the provisioning behavior of others, resulting in uncertainty about the relative contributions of other carers and the current needs of the offspring (Johnstone and Hinde 2006). Both of these factors influence the provisioning decisions of carers, and the level of uncertainty becomes progressively higher as greater numbers of individuals contribute to care.

Existing theory on offspring care has focused on the total amount of care delivered and on the response of carers to a change in the amount delivered by others. Models have typically adopted either “sealed bid” (Houston and Davies 1985; Savage et al. 2013) or “negotiation” (McNamara et al. 1999; Johnstone 2011; Lessells and McNamara 2012) approaches to determine the optimum investment level (or response rule, in negotiation models) for each carer during a breeding attempt. Both methods produce qualitatively similar results, generally predicting incomplete compensation to changes in investment by other carers (McNamara et al. 1999), although additional considerations such as asymmetric information about the offspring among carers (Johnstone and Hinde 2006), maternal tactics (Savage et al. 2013), or threshold effects such as partner desertion (Jones et al. 2002) can lead to alternative predictions. Empirical work supports incomplete compensation as the usual strategy adopted in biparental species (although with substantial variation, reviewed in Harrison et al. 2009), but results are more mixed in cooperative species where non-breeding individuals also contribute to care (Hatchwell 1999).

The solutions to sealed-bid and negotiation models do not generally lead to the best possible outcome for carers as a group, since their joint payoffs could be increased by all carers simultaneously raising their investment (Johnstone et al. 2014). When individuals are highly responsive to changes in investment by others, negotiation may even result in offspring receiving less investment from two cooperating parents than from a lone parent (Royle et al. 2002; McNamara et al. 2003). How might animals avoid these negative outcomes of negotiation? Existing work suggests that efficient cooperation can occur if repeated interactions allow individuals to reward cooperators and/or police exploiters/defectors (Axelrod and Hamilton 1981; Frank 2003), relying on simple mechanisms such as group knowledge of the contributions provided by others (Keser and Van Winden 2000). The tendency of individuals to invest more in a common project if others invest similarly is known from human studies to assist in optimally providing for a common good (Fischbacher et al. 2001; Frey and Meier 2004), but evidence from animal studies is contentious (Raihani and Bshary 2011). It may be that limitations in cognition restrict animals from adopting such strategies by hampering their ability to track the contributions of others (West et al. 2007; McAuliffe and Thornton 2015).

In many natural cooperative care systems, investment in offspring takes the form of collective provisioning by all carers, which is split into many discrete events wherein a single carer brings food to the offspring. These repeated events provide an opportunity for individuals to minimize potential “cheating” by applying a “turn-taking” strategy to offspring care: if individuals reduce their provisioning rate after they visit, and increase it after others visit, carer contributions will be approximately fair over the breeding attempt. Such strategies are not available in classic negotiation models that do not allow individuals to respond to individual provisioning events by their partners, but such rules are behaviorally and cognitively simple and thus biologically feasible. Perfect turn-taking is unlikely to occur in natural systems due to imperfect information, individual differences, and stochastic factors; however, any significant response to other carers will improve investment efficiency (Johnstone et al. 2014). Empirical work on great tits (*Parus major*) has provided evidence that such reciprocal investment rules are used to regulate provisioning of young (Johnstone et al. 2014) and in pair-breeding long-tailed tits (*Aegithalos caudatus*); higher degrees of turn-taking are associated with higher food delivery rates and lower predation risk (Bebbington and Hatchwell 2016).

The provisioning rules of cooperative breeding systems, where non-parents also help to rear offspring, provide an interesting contrast to systems with only two carers. With larger numbers of carers, coordinating nest visits may become increasingly important to avoid one or more individuals contributing disproportionately. Turn-taking rules also become more difficult to implement, as carers must track the contributions of more individuals, potentially leading to the adoption of simpler response rules. In addition, helpers in cooperative systems can vary in quality or condition (Clutton-Brock et al. 2002) and might contribute to care for a variety of reasons that could lead to different provisioning rules. The presence of turn-taking rules during cooperative provisioning has hitherto been investigated in two species: acorn woodpeckers (*Melanerpes formicivorus*), which exhibit a strong tendency to alternate visits (Koenig and Walters 2016), and riflemen (*Acanthisitta chloris*), which do not (Khwaja et al. 2017). As both these studies assess turn-taking solely in terms of visit order, a logical next step is to implement more complex analyses incorporating the timing of visits and investigating whether particular classes of individual (e.g., helpers) are more responsive or more likely to take turns.

Here, we modify the approach of Johnstone et al. (2014) to investigate turn-taking in a species with more than two carers. We analyze nest provisioning data from the cooperatively breeding chestnut-crowned babbler (*Pomatostomus ruficeps*), a medium-sized (50 g) endemic Australian passerine that breeds in groups of 2–15 (mean ≈ 6), with helpers provisioning at the nest in addition to the breeding pair (Russell et al. 2010; Browning et al. 2012a). Babbler breeding success is closely related to the number of carers in the group (Liebl et al. 2016), and coordination of care is likely to be important as breeding is costly: all individuals in the group lose mass as breeding events progress (Sorato et al. 2016). Babbler groups provide an appropriate system to investigate fine-scale response rules in the provisioning of offspring, as they forage together within stable home ranges (Portelli et al. 2009; Nomano et al. 2014), do not “false feed” when delivering food to offspring (Young et al. 2013), and helpers seem to be concerned largely with accruing kin-selected benefits rather than social prestige (Browning et al. 2012a; Nomano et al. 2013). While babbler breeding groups normally move around their territories as a cohesive unit, provisioning does not occur as a group: individual carers do not feed every time the group is near the nest, but may forage for food to deliver after other birds provision, or (more rarely) feed twice in relatively quick succession if the group remains nearby for an extended period (Nomano et al. 2014; Sorato et al. 2016), providing scope to respond to the investment of other group members.

Our primary aim in this article is to investigate whether turn-taking occurs within groups of babblers provisioning offspring and, if so, to investigate whether it can be attributed to individuals directly responding to each other. Secondarily, we are investigating differences between types of carer (breeding female, breeding male, and helpers ranked by visit rate) in their propensity for turn-taking. To this end, we generate and fit models of provisioning behavior using a continuous-time Markov-Chain Monte-Carlo (MCMC) approach (Bremaud 2001; Harcourt et al. 2010) and apply a randomization test to distinguish direct responsiveness (“active” turn-taking) from alternating visits caused by other effects (“passive” turn-taking). Markov-Chain models have a long history of being applied to animal behavioral sequences (Cane 1959), and a continuous-time approach is required as individuals can visit the nest at any time. Such models have previously been used with success to describe animal behavioral rules in several contexts (Harcourt et al. 2009, 2010; Patterson et al. 2009; Johnstone et al. 2014), and we demonstrate here that they are also applicable to studying the fine-scale organization of provisioning in cooperative breeders.

## Methods

### Data collection

Provisioning data were collected between July and November in both 2007 and 2008 at Fowlers Gap Arid Zone Research Station, NSW, Australia (Lat. − 31.1, Long. 141.7). Adults were caught using mist-nets, ringed, tagged with Passive Integrated Transponder (PIT) tags (2 × 12 mm, Trovan Ltd.), and had a small (< 100 μl) blood sample taken from the ulnar vein. Chicks were similarly blood-sampled, ringed, and PIT-tagged at around 15 days old. Chestnut-crowned babblers are sexually monomorphic, so identification of parentage and sex was primarily obtained through molecular analysis (Holleley et al. 2009; Rollins et al. 2012). Group composition was determined by repeated counts and color-ring sightings before and after group capture, and only groups with all individuals PIT-tagged were analyzed. Chestnut-crowned babblers build large enclosed stick nests with a single entrance, around which we fitted a coil antenna connected to a data-logger (LID650, Dorset ID b.v.) at the base of the nest tree; this allowed us to precisely monitor all nest visits by PIT-tagged individuals. For further details of the system and methods used to collect nest visit data, see Young et al. (2013).

For our analysis, we restricted ourselves to nest visit data from broods older than 10 days, to avoid confusing female brooding behavior with provisioning, and to datasets in which all significant carers (see below) visited more than ten times each. We also discounted data from the last day in the nest prior to fledging (typically days 20–22) and any data from periods of disturbance (such as periodic measuring of chicks or changing of data loggers). We grouped multiple PIT records by the same individual into a single visit when they occurred within 2 min, an approach validated by previous studies on babbler provisioning (Browning et al. 2012a; Nomano et al. 2014). As all behavioral data were collected using automatic data-loggers, the study methods were intrinsically blind.

### Classification of carers

In chestnut-crowned babblers, some individuals in a group do not visit the nest to assist with chick provisioning or visit infrequently; these individuals are usually immigrants or juveniles (Browning et al. 2012a). For our purposes, we are interested only in significant carers, not total group size, as individuals that contribute rarely to offspring care have little opportunity to respond to the investment of others. Furthermore, attempting to fit individuals with very few visits into our model is problematic because some possible state transitions may never appear in the data (e.g., two rare visitors may, purely by chance, never follow one another), making it impossible to estimate some theoretically viable visit rates. To determine the number of significant carers, individuals within each group were first ranked in order of total number of visits. The individual with the least visits was then excluded if it failed to exceed 20% of the mean number of visits by the rest of the group (e.g., a bird with ≤ 10 visits would be excluded in a group comprising four other birds that each visited 50 times). If excluded, the process was repeated with the next-lowest visiting individual until the least-visiting individual exceeded 20% of the mean of the other individuals in the group. Below, helpers are differentiated from each other purely by their visit rate, with the helper visiting the most referred to as the primary helper.

Applying the above process to all groups with complete visit rate, data available over several days resulted in four groups with two (significant) carers, six with three carers, five with four carers, three with five carers, and two with six carers; between zero and five, individuals in each group were excluded for failing to classify as a significant carer (mean = 1.1). The amount of data required to fit our models grows rapidly as group size increases, because the number of transitions to be estimated is equal to the square of the carer number. This escalating need for data at large group sizes meant that two groups with good provisioning data were excluded from the final analysis because they contained too many significant carers (eight and nine) to fit our models.

Among the groups used, brood size ranged from 1 to 5 chicks (mean 3.3), and chick age from 10 to 19 days old; previous work suggests that babbler provisioning rate is relatively static between day 10 and day 20 (Browning et al. 2012b). Differences in provisioning rates between different types of carer and effects of nestling age have previously been described for this system in detail and with higher sample sizes (Browning et al. 2012a, b; Nomano et al. 2015), so we did not pursue any further analyses of the relative levels of contribution provided by different carers. The datasets analyzed during the current study are available from the corresponding author on reasonable request.

### Distinguishing passive and active turn-taking

To analyze turn-taking, it is first necessary to characterize how many alternated visits one would expect among individuals visiting randomly with no regard for each other’s behavior: we refer to these chance alternations as passive turn-taking. Most obviously, carer number will strongly influence the expected proportion of alternated visits, as individuals in larger groups contribute a smaller proportion of the total number of visits and so are less likely to visit twice in a row by chance. In addition, the more unequal the distribution of visits among individuals, the more likely it is for the rarer individual to alternate its visits. We used a k-category runs test (Sheskin 2011) that accounts for both these effects as an initial test to assess if the number of alternated visits differed from that expected by chance for groups of different sizes and visit rate distributions.

A second problem with naïvely analyzing visit data for patterns of turn-taking is that additional alternated visits can arise from the manner in which individuals’ provision. For example, if each individual must spend a certain amount of time foraging and traveling to and from the offspring between successive provisioning events, then additional passive turn-taking will arise simply because other individuals can visit during this interval in which the focal bird cannot. If the minimum inter-visit interval (IVI) of each individual is highly consistent, near-perfect turn-taking (or a regular pattern, if visit rate varies between individuals) might be expected even if individuals do not monitor or respond to one another’s behavior in any way. This turn-taking will be less precise if this “refractory period” is variable, but even with highly variable intervals, any significant refractory period will introduce some additional passive turn-taking. Furthermore, continuous-time Markov models assume that events (IVIs, in this case) are approximately exponentially distributed, an assumption violated when significant refractory periods exist. One possible solution is to fit a more complex semi-Markov model explicitly defining the event distribution; however, we lacked sufficient data on individual, group, and environmental variation in IVIs to fit this model effectively. To circumvent these problems and fully distinguish between passive turn-taking and active turn-taking, we therefore employed a randomization test similar to that of Johnstone et al. (2014).

Our randomization test removes any potential active turn-taking from the data by eliminating relationships between the visit times of different individuals. We first calculated all of an individual’s IVIs in a given day, then randomly re-ordered these intervals within each individual and day. From these randomized intervals, we reconstructed a new list of artificial visit times for each individual on each day. The artificial visit times for all individuals were then combined to form an artificial provisioning day, which could then be analyzed in the same way as the original data. We re-ran the above dissociation 1000 times on the data from each breeding attempt to generate distributions of expected (passive) turn-taking given the structure of nest visits within each attempt. By comparing the extent of turn-taking observed in the natural data to the distribution of values obtained from the dissociated data, we can determine the degree to which turn-taking is passive, i.e., simply attributable to group size, unequal visit rates, and a lack of immediate re-visits, or active, being caused by individuals responding to one another’s behavior. This type of randomization test can be biased if individual IVIs are ordered across the observation period (Schlicht et al. 2016), so the degree of order was estimated before data were randomized.

One final consideration is that individuals adopting a group foraging strategy will be associated with each other simply because they may tend to deliver food to chicks at the same time. This might contribute to turn-taking by increasing the effective refractory period or by raising the likelihood that multiple different individuals provision in quick succession. While a full exploration of the effects of different group foraging strategies is beyond the scope of this study, an important consideration is that our assumptions about individuals being able to respond to the investment of others would be violated if groups adopt strict “bouts” of highly synchronous provisioning during which most or all group members feed the chicks. To confirm that our assumptions were valid, we first calculated group IVIs for the first full day of provisioning following decoder setup at each nest. If group (larger than two) provision in synchronous bouts, their IVIs will tend to cluster, with more consecutive short IVIs than would be expected by chance. Whether short IVIs are significantly clustered can hence be tested using a standard Wald-Wolfowitz runs test, after categorizing each IVI as either short or long based on a threshold value. Fewer runs than expected would indicate clustering and suggest some degree of group-level provisioning.

### Markov analysis

**Fig. 1:**
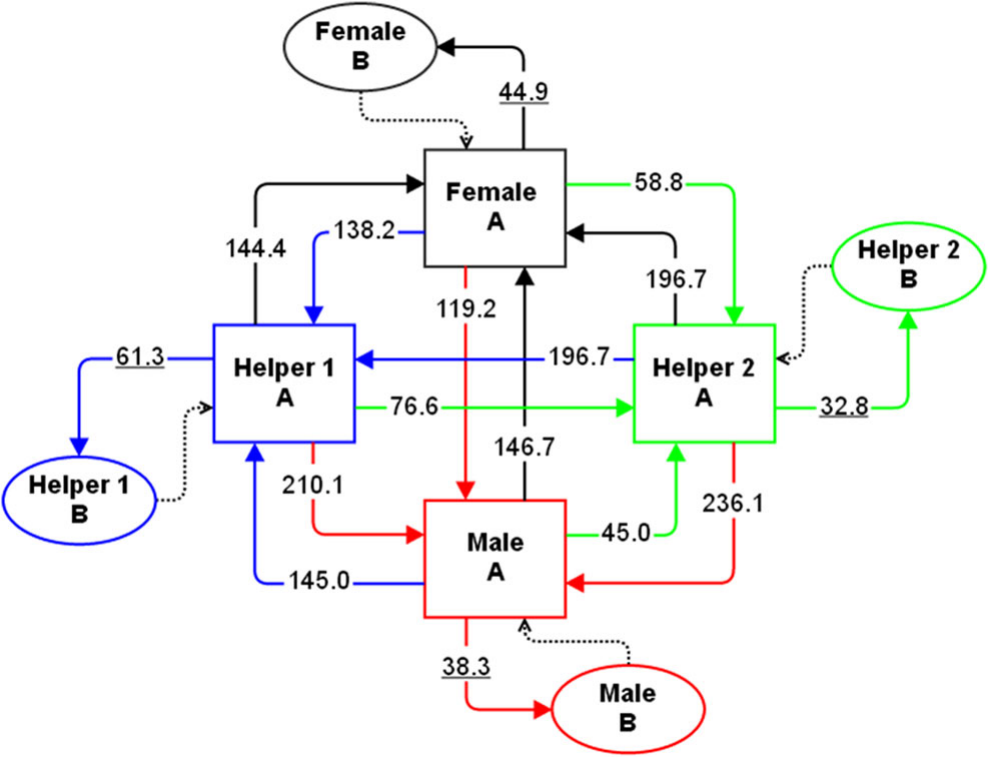
An illustrative Markov model of provisioning in a cooperative group with four carers. There are eight possible states, two (A and B) for each carer, and 16 possible transitions (solid arrows), which represent the current provisioner visiting after either itself or each of the other three carers. Each time a bird provisions the model moves to a new state along a particular transition depending on the identities of the current and previous provisioner. The dashed arrows represent the automatic transitions that reset carers to their base state (A) immediately after they have made a repeat visit. The values attached to each transition show the estimated transition rates for a particular four-bird group; within individual transition, rates (*μ*) are underlined

For each breeding unit, we fitted a continuous-time Markov chain model to the nest visits using the R package msm (Jackson 2011). Each visit was treated as a discrete event, and the model “state” was defined by the identity of the last individual to visit the nest. This formulation violates the assumptions of msm, because some events, specifically repeat visits by the same bird, do not change the state of the system. To allow for this, we added a second “dummy” state for each bird to represent a repeat visit by the same individual. Whenever a transition to a dummy state occurred (i.e., whenever there was a repeat visit), we imposed an immediate “reset” transition to return the system to the base state for that individual. Figure 1 displays an example model for a four-bird group. With this arrangement, which we used in all of our analyses, groups with *c* carers featured 2*c* states and *c*(*c* + 1) possible transitions between states, including reset transitions. The best-fit model calculated by msm specifies the rate at which each possible transition occurs (which determines how likely particular birds are to follow particular other birds). We assume that individuals may react differently to each of the other carers visiting the nest and thus estimate, for each individual, as many different visit rates as there are carers, giving *c* ^2^ transition (visit) rates to be estimated (resets are fixed to an arbitrary, very high rate).

Owing to the numerous possible transitions in larger groups, it is also useful to define an individual’s visit rate when they follow any bird other than themselves. Following Johnstone et al. (2014), we define this visit rate (“following another”) as *λ*_*i*_ for each individual and the visit rate when an individual makes a repeat visit (“following self”) as *μ*_*i*_. To fit this model, we constrain the analysis such that visit rates when following any other bird are equivalent and compare whether this reduced model (with 2*c* unique transitions) explains the observed data similarly well compared to the full model. Similarly, we can further constrain the analysis to model all individuals visiting at the same rate, giving only two possible transition rates regardless of group size: any bird following any other bird (*λ*_0_) and a bird following itself (*μ*_0_). Comparisons between models were made using likelihood ratio tests, which follow a chi-squared distribution with degrees of freedom equal to the difference in free parameters between the full and constrained models.

## Results

### Visit rates and turn-taking ratios

**Fig. 2:**
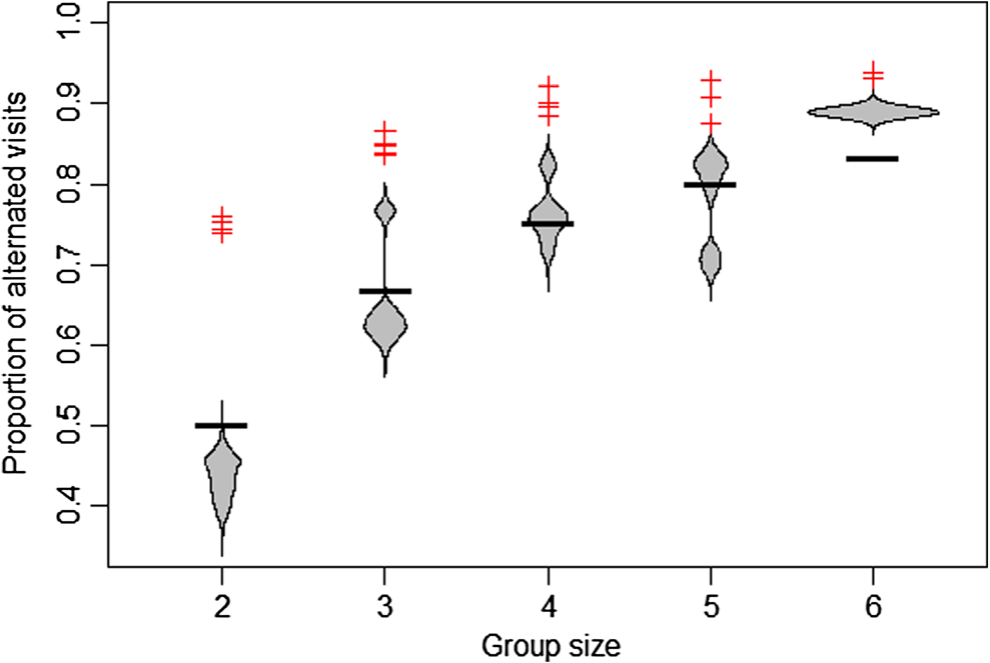
Proportion of alternated visits (those following another bird) vs. repeat visits for different group sizes. Natural data (red crosses) indicate that the proportion of alternated visits increases with group size, as expected, and is consistently greater than the amount of alternation expected purely due to the number of individuals (black bars). Beanplots show the expected proportion of alternated visits in natural data (1000 randomizations of individual inter-visit intervals per group), suggesting that the observed rates of alternation cannot be solely attributed to group size, variation in visit rate, or the distribution of individual inter-visit intervals. Smaller groups show greater deviation from randomized values than larger groups

Provisioning data were analyzed for 20 breeding attempts by 19 different breeding groups (315–1475 nest visits per attempt, mean = 765). Across all carers (*n* = 73) and group sizes (2–6), individuals alternated visits (i.e., followed another bird) more often than they visited twice in a row, and as expected, the proportion of alternated visits increased with group size (range 0.75–0.94 for group sizes 2–6, mean proportion across all attempts 0.85) (Fig. 2). The number of alternated visits was greater than expected for all 20 breeding attempts (k-category runs tests, all *p* < 0.001).

**Fig. 3:**
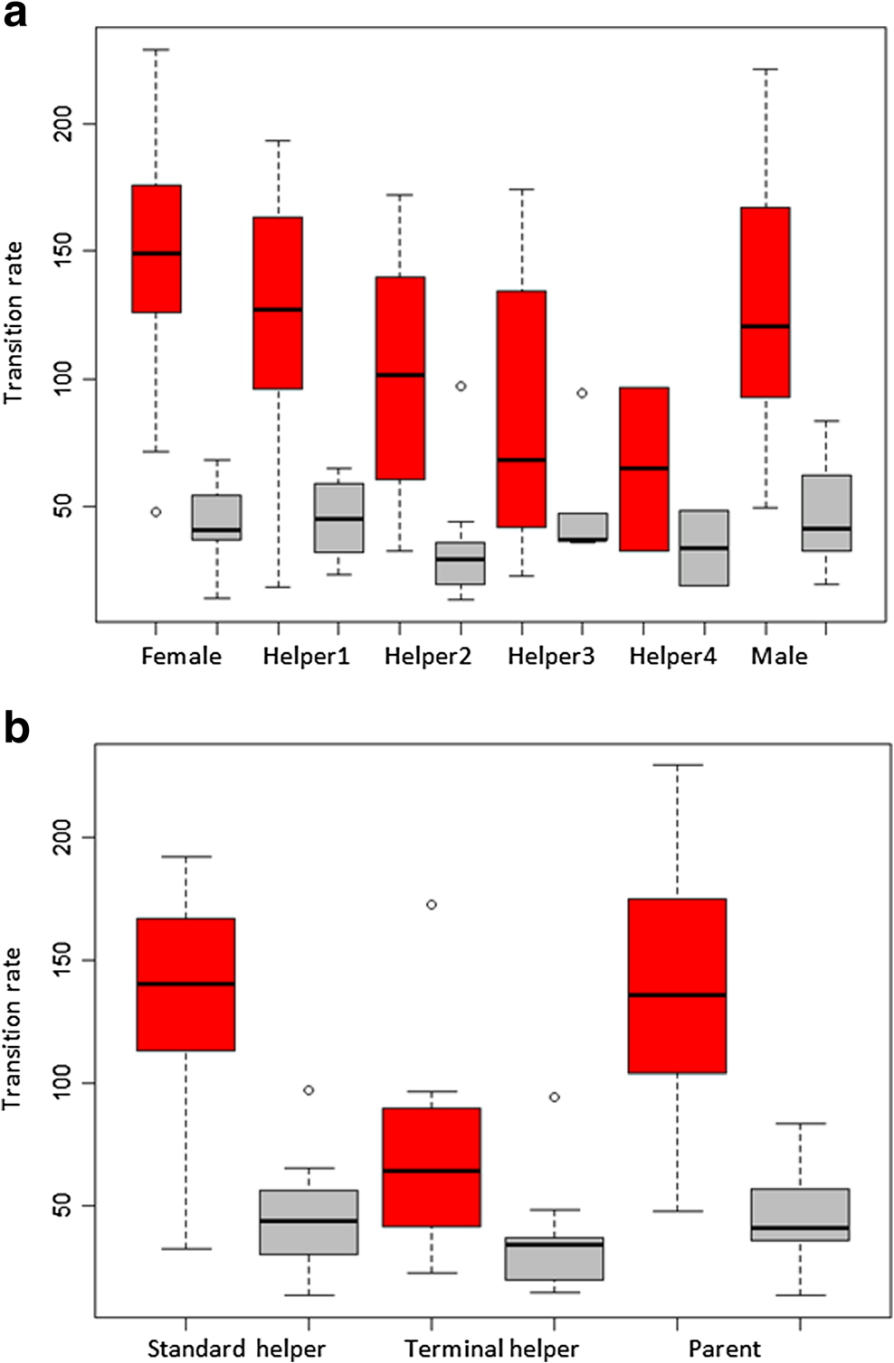
**a** Individuals are generally more likely to follow other birds than to visit the nest twice in a row, demonstrating turn-taking in the provisioning of offspring. Turn-taking is strongest for breeding males, females and the primary (most helpful) helpers and then decreases with helper visit rate. **b** Although all types of carer show a tendency to alternate, differences in visit rate arise between terminal (lowest visit rate) helpers when compared to the other helpers contributing to that breeding attempt (“standard” helpers). Standard helpers and parents are similar in their visit rate and proportion of alternated visits. Box plots indicate between-individual visit rates (red) and within-individual visit rates (gray); boxes indicate the median ± quartiles, and whiskers extend to the most extreme data point that is within 1.5 times the interquartile range of the box

Markov models were then fitted for each breeding attempt according to the number of carers; Fig. 1 shows an example fitted model for a four-bird group. Our Markov analysis further supported the occurrence of turn-taking, as across all carers, the rate at which birds made alternated visits (*λ* = 123.7 ± 43.4) was much higher than the rate at which they made repeated visits (*μ* = 50.5 ± 18.2) (mean ± SD, Wilcoxon test, *p* < 0.0001). One breeding attempt was excluded from further analysis due to insufficient data to fit individual-specific models; for a majority (17/19) of the remaining groups, models in which individuals visited at different rates following particular other individuals were a better fit (likelihood ratio test, *p* < 0.05) than models that assumed each individual had only two possible visit rates (*λ*_*i*_ and *μ*_*i*_). In all groups, both of the above models were much better fits to the data (likelihood ratio test, *p* < 0.01) than a null model assuming all individuals had the same two possible rates (*λ*_0_ and *μ*_0_). Despite the significance of individual differences in transition rates within groups, across all groups, neither within-individual nor between-individual visit rates were significantly different between breeding males, breeding females, and the primary (most helpful) non-breeding helpers in a group (Fig. 3a). Likewise, classes of carer did not differ in their tendency to follow other classes (e.g., helpers vs. breeding males following the breeding female). The only significant differences among classes of carers were between the lowest visit rate helper and the other carers in groups with more than one helper (Fig. 3b).

There were no significant influences on individual transition rates from mean visit rate of the breeding group, group size, brood size, brood age, or the number of carers per offspring (*p* > 0.05), although this may be attributable to small sample sizes and our restricted window of analysis, as some of the above factors are known to influence babbler provisioning behavior when the entire rearing period is considered (Browning et al. 2012b). There was also no significant pairwise relationship between individual transition intensities, suggesting that particular birds were not more likely to follow birds that in turn were more likely to follow them. We infer from this finding that there are no sub-group units within which babblers adopt turn-taking rules.

### Inter-visit intervals

As indicated above, analysis of natural data shows that individual babblers are less likely to visit again after they have just visited themselves. This turn-taking could be attributed to individuals actively responding to investment by others or be caused by passive constraints on the distribution of individual IVIs, for instance because individuals require a well-defined minimum period to forage for food items and return to the nest. Median individual IVI across different group sizes and carer categories ranged between 6.55 and 10.18 min (means 8.70–22.07 min), forming an approximately geometric distribution with a long tail of larger intervals consistent with the absence of a refractory period (aside from the 2 min minimum interval created by the initial processing of the PIT data: see methods). Approximately 97% of all IVIs were less than 1 h, suggesting that all individuals visited throughout the day. As might be expected, the intervals between any one carer visiting the nest were smaller in larger groups (mean ± SD of median intervals 3.03 ± 0.46 min for pairs and trios, 1.98 ± 0.69 min for larger groups). Histograms of individual IVIs for different group sizes are included in supplementary material (Fig. S1). There was no indication that individual IVIs were ordered with respect to length (mean ± SD of individual *p* scores 0.507 ± 0.022; Fig. S3), supporting the validity of our randomization test (Johnstone et al. 2016; Schlicht et al. 2016).

### Passive or active turn-taking?

**Fig. 4:**
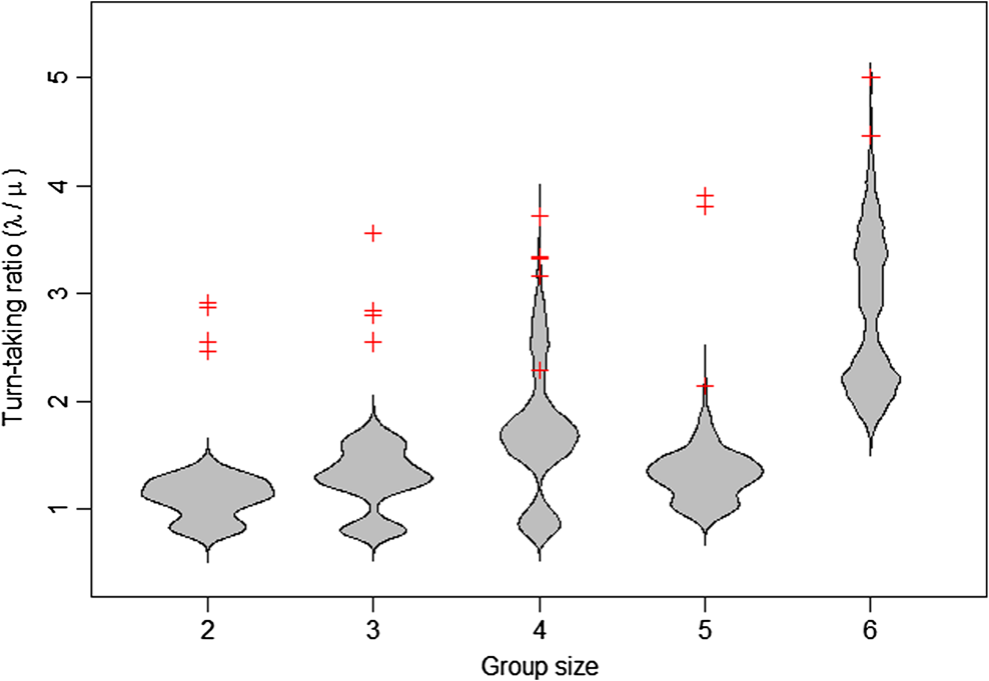
In all babbler groups analyzed, the observed mean transition rate following other birds (*λ*) was greater than the rate of repeat visits (*μ*); the *λ*/*μ* ratio did not change significantly across group sizes (crosses). Beanplots show the expected *λ*/*μ* ratio if individuals were not responding to the investment of others. Beanplots were generated by randomizing individual inter-visit intervals 1000 times (per group) and then re-analyzing using the same method as the natural data. For all groups, natural data showed significantly greater turn-taking than randomized data

Our randomization analysis supported the existence of both passive and active turn-taking, as the natural data exhibited a higher proportion of alternated visits than expected by chance (Fig. 2). In addition to the greater frequency of alternation in the natural vs. randomized data (Fig. 2), the tendency for individual babblers to exhibit turn-taking (*λ*/*μ* > 1) in fitted Markov models was far lower under our randomization test than in the natural data (Fig. 4). Of the 19 breeding attempts analyzed, randomization tests generated turn-taking rates that overlapped with those in the natural data in only a single group. Nevertheless, significant passive turn-taking is still present in a majority of groups in the randomized data (12/19 groups exclude *λ*/*μ* = 1 from the range of 1000 randomized data runs), suggesting that individual IVIs generate some degrees of visit structure. Individual IVIs were randomly ordered for every individual in our dataset (mean *p* score = 0.507, range = 0.467–0.571; Fig. S2), suggesting that the randomization was unbiased (Schlicht et al. 2016).

There was no strong indication that group size affected the degree of turn-taking by individual birds (Fig. 4), and we found no evidence for group bouts of provisioning during which all individuals feed (runs test of group IVIs using median threshold, all *p* > 0.05) or any group visit patterns inconsistent with a broadly random distribution of nest arrival times under occasional disturbance (Fig. S3).

## Discussion

We found strong evidence that chestnut-crowned babblers respond to other carers visiting the nest by modifying their own visit rate (active turn-taking), beyond the apparent turn-taking arising from combination of group size effects, unequal visit rates, and the distribution of individual inter-visit intervals (passive turn-taking). Babblers exhibited active turn-taking across all group sizes, and all carers with similar visit rates adopted similar provisioning rules. We found no evidence that the observed levels of active turn-taking could be attributed to synchronous nest visits by the care group.

Both our run tests and Markov analyses suggested that some of the tendencies for individuals to take turns could not be explained by group size effects alone. Refractory periods are a likely cause for some of this additional passive turn-taking, as individual carers may take some well-defined minimum period to travel to and from the foraging site and another minimum period to find food while foraging. Consequently, individual IVIs will have a well-defined minimum value, and longer minimum IVIs will arise whenever carers cease provisioning chicks to collect food for themselves. Under these circumstances, some degrees of turn-taking will always occur because during the refractory period, other carers can visit while the focal individual cannot. For the above reason, some passive turn-taking was inevitable in our data due to the way the raw PIT-tag data was processed: no individual IVI could be less than 2 min, as PIT readings from the same individual were grouped into a single visit if they occurred within 2 min of each other. While the choice of a 2-min grouping window has been validated using nest video data and nest-watches (Browning et al. 2012a), and consequently we believe our results to be robust, this pre-processing makes it difficult to precisely assess how much passive turn-taking occurs due to natural refractory periods in babblers.

Similarly, differences in individual visit rates can also increase or decrease the amount of passive turn-taking in natural data. While we found no consistent differences in care behavior between males, females, and primary helpers, individually parameterized Markov models were supported in the majority of groups, suggesting that individual visit rates influence turn-taking metrics. This makes intuitive sense, as an individual visiting more regularly than others in its group will be followed more by every other group member. Conversely, an unequal distribution of visits depresses the expected proportion of alternated visits, potentially explaining why randomized data from pairs has a median below the expected value of 0.5 (Fig. 2).

Several possible reasons for babblers exhibiting both active and passive turn-taking (beyond that attributable to group size) are suggested by their breeding behavior and ecology. Compared to great- and long-tailed tits, babblers forage more distantly from the nest (Sorato et al. 2016), are not agile flyers, and appear to suffer costs associated with traveling to and from the nest (Browning et al. 2012b). These factors decrease the ability of individual babblers to deliver separate food items in quick succession and will hence bias the natural data towards passive turn-taking. Babblers do not false feed (Young et al. 2013) and—unlike great tits—show more variability in visit rate than in the size of prey they deliver (Browning et al. 2012b), supporting the use of simple turn-taking rules as visit rate alone is a reasonable proxy for investment delivered. Acorn woodpeckers have similar mean group sizes and mean individual IVIs to babblers (Koenig and Walters 2016), making it feasible that their observed turn-taking is likewise a combination of passive and active alternation. Riflemen remain the only published example of a cooperative species without a clear signal of turn-taking; one possibility is that the low levels of sexual conflict in this species minimize the risks of being exploited during provisioning (Khwaja et al. 2017). Babblers forage as a group and hence often return to the nest area together (Sorato et al. 2012; Nomano et al. 2014); however, we found no evidence that babblers visit immediately after each other in strict bouts. It remains unclear how potentially complex foraging behavior in large groups might affect both apparent passive turn-taking and the information birds have about the contributions of others.

Theoretical and empirical work suggests that turn-taking is an efficient way to organize investment in a brood of young (Johnstone et al. 2014); however, species will vary in their ability to adopt a provisioning rule that requires attending to the contributions of others. Whether individuals cooperating to rear young will adopt turn-taking rather than applying certain alternative strategies of “negotiation” (McNamara et al. 1999, 2003; Johnstone 2011) or “sealed bids” (Houston and Davies 1985) at the start of the breeding attempt seems likely to depend on the biology and feeding ecology of the species in question. If the costs of acquiring the necessary information about group investment are high, and the accuracy or benefit of obtaining such information low, an individual may do better to make investment decisions based only on its knowledge of the brood and its current personal energy reserves. Alternatively, a helper may contribute for direct benefits such as breeding experience, in which case the behavior of other carers has little bearing on its investment choices. Conversely, cooperative species seem likely to adopt an alternation rule to organize their investment in offspring when contributions to care are easily observable by the entire group.

Given the increasing ease with which large datasets on parental care contributions can now be collected on wild populations, there is substantial scope for future studies on fine-scale patterns of parental care. Of particular interest would be how patterns such as turn-taking and visit synchrony respond to experimental manipulation, and whether these responses differ across species with different levels of sexual (or parent-helper) conflict (Mariette and Griffith 2015; Khwaja et al. 2017). These patterns may also interact, depending on the biology of the species in question. For example, if adopting a turn-taking rule is beneficial and easier to implement when carers visit the nest together, turn-taking might provide an additional explanation for the high visit synchrony observed in several bird species (Marzluff and Balda 1990; Doutrelant and Covas 2007), which is often associated with increased breeding success (Raihani et al. 2010; Mariette and Griffith 2012; Bebbington and Hatchwell 2016). More research is needed to determine the prevalence of turn-taking outside of the few species hitherto studied, and the degrees to which the potential benefits of turn-taking are realized in nature.

## Electronic supplementary material

ESM 1 (PDF 333 kb)
