# Supplementary material for: Turn-taking in cooperative offspring care: by-product of individual provisioning behavior or active response rule?
Source: Behav Ecol Sociobiol. 2017 Oct 17;71(11):162. doi: 10.1007/s00265-017-2391-4 (PMC5644705; doi:10.1007/s00265-017-2391-4)
Supplement: Supplementary file 1 — (PDF 333 kb) [file 265_2017_2391_MOESM1_ESM.pdf]

Turn-taking in cooperative offspring care: by-product of individual provisioning behavior or active response rule? (Supplementary Figures)

James L. Savage[1], Lucy E. Browning, Andrea Manica, Andrew F. Russell & Rufus A. Johnstone

[1]Corresponding author: james.savage@cantab.net

Department of Zoology, University of Cambridge; Department of Animal Sciences, Wageningen University; School of Biological, Earth and Environmental Sciences, University College Cork.

Behavioral Ecology and Sociobiology

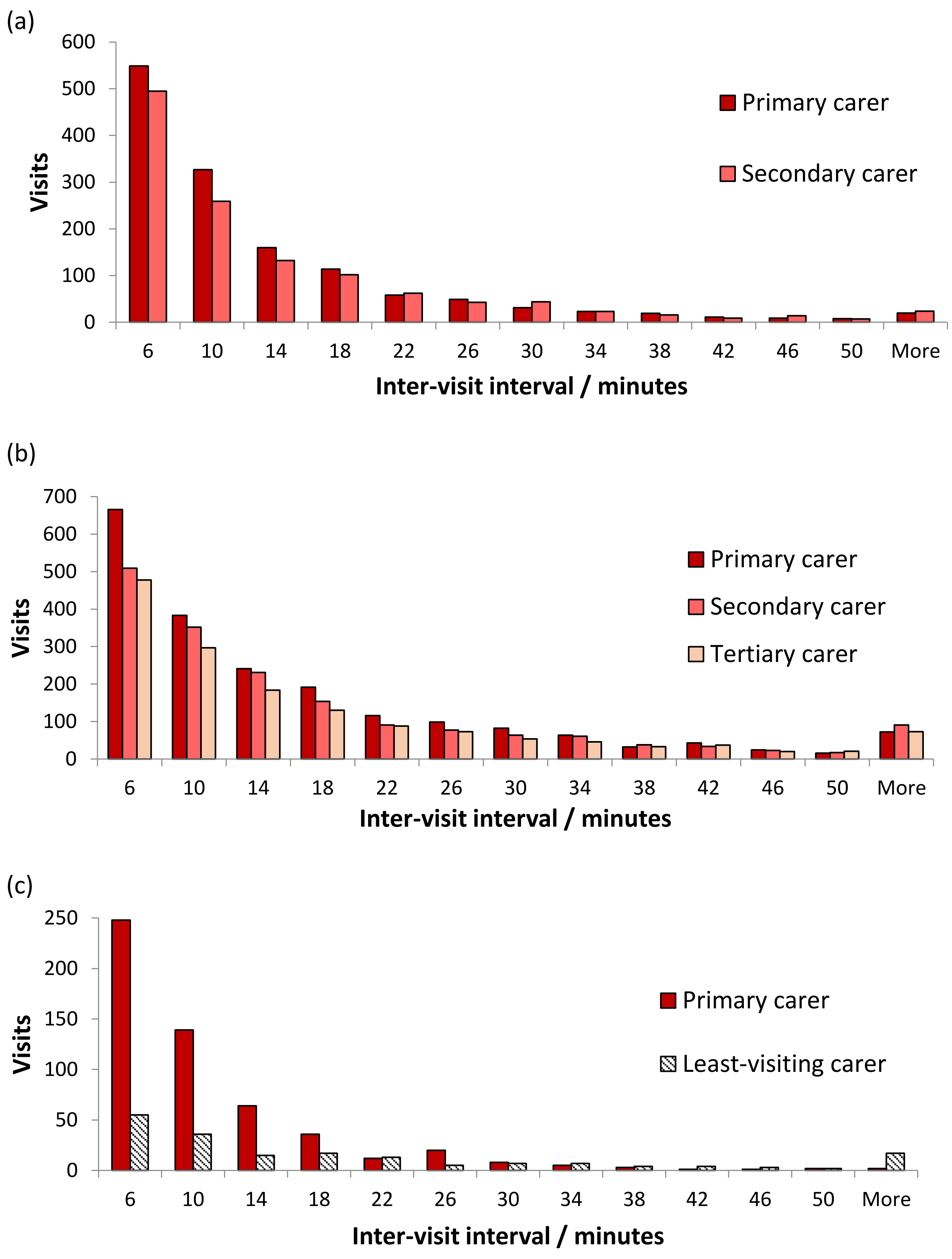

Figure S1: The distributions of individual inter-visit intervals are similar for all classes of carer. In groups with only two (a) and three (b) significant carers, the only differences between carers which help most and carers which help least is in the visit rate, i.e. a given visit from any individual is equally likely to be followed by a short interval as a long interval. In larger groups the aforementioned difference between terminal helpers and other carers appears (c): the visits of the individual that visits least in 6-carer groups are much more likely to occur at long intervals than those of the primary carer.

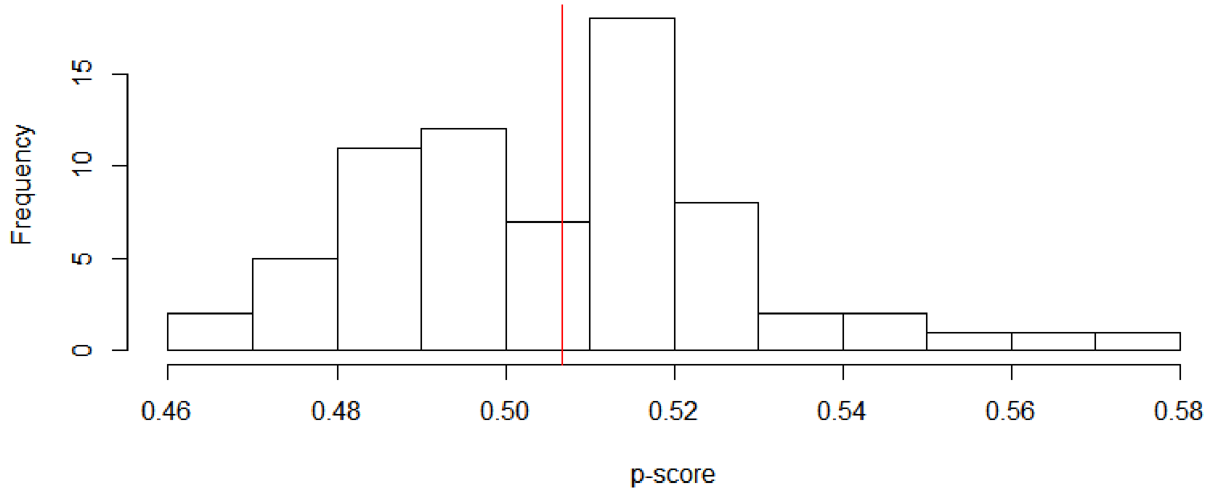

Figure S2: Histogram of *p*-score (a measure of degree of ordering, Schlicht et al. 2016) of individual inter-visit intervals of all birds included in our study. Ordered visits would potentially bias our randomisation tests, leading to erroneous conclusions about the degree of active turn-taking. All individuals score very close to 0.5, indicating that their intervals are randomly ordered (1 = strictly increasing, 0 = strictly decreasing). Mean score across all individuals is indicated by the red vertical line.

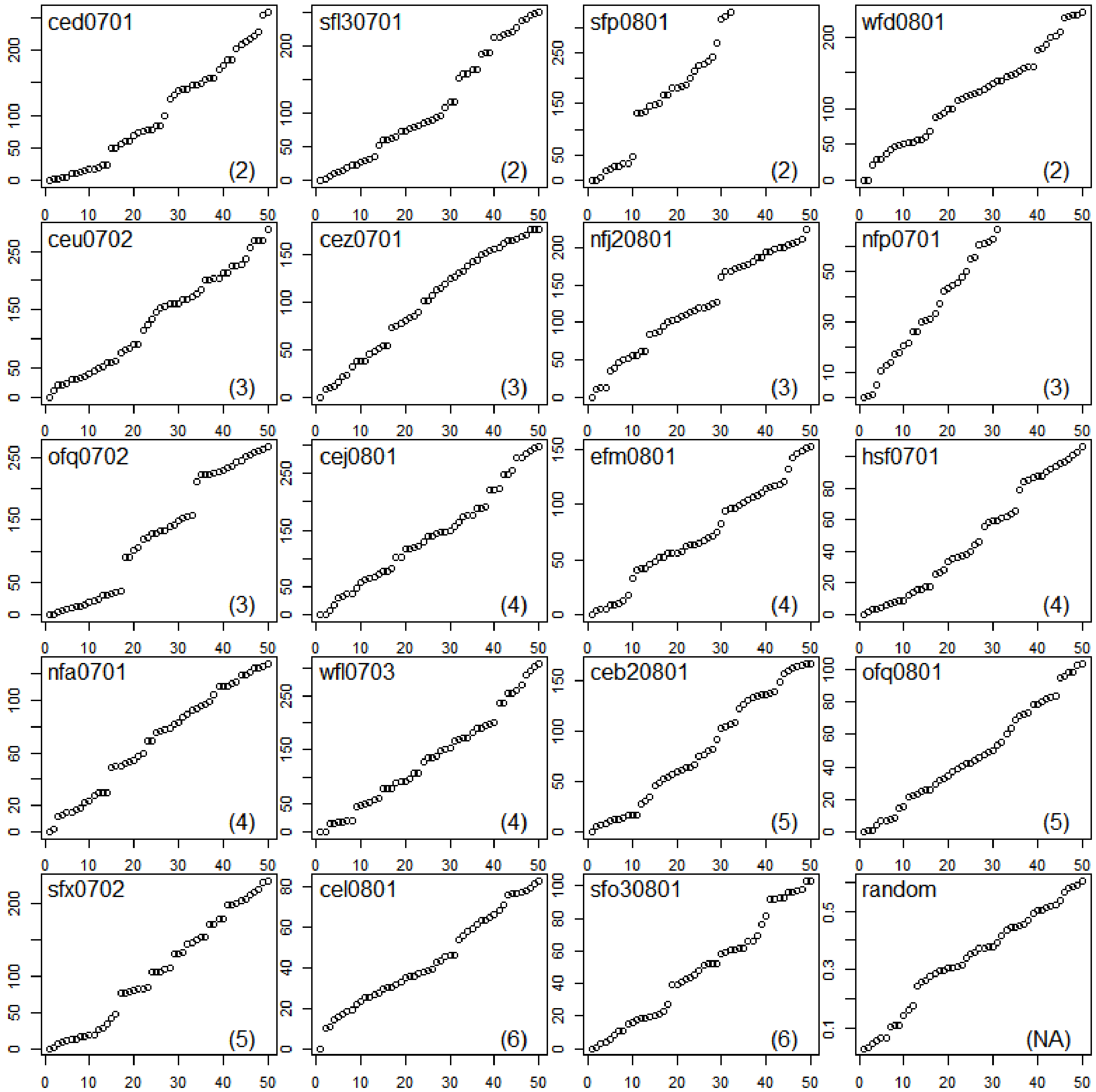

Figure S3: Sample timeline of nest visits for each nest included in our analysis (n=19) and sorted random arrival times (sampled from a uniform distribution) for comparison. Provisioning shows no clear evidence of synchronous bouts of feeding by the entire group. Data plotted are the first 50 nest visits in the first day after decoder setup. The number of significant carers is given in the bottom right of each plot.
